# A Wearable Breath Sensor Based on Fiber-Tip Microcantilever

**DOI:** 10.3390/bios12030168

**Published:** 2022-03-07

**Authors:** Cong Zhao, Dan Liu, Zhihao Cai, Bin Du, Mengqiang Zou, Shuo Tang, Bozhe Li, Cong Xiong, Peng Ji, Lichao Zhang, Yuan Gong, Gaixia Xu, Changrui Liao, Yiping Wang

**Affiliations:** 1 Key Laboratory of Optoelectronic Devices and Systems of Ministry of Education and Guangdong Province, College of Physics and Optoelectronic Engineering, Shenzhen University, Shenzhen 518060, China; zhaocong@szu.edu.cn (C.Z.); 2150120415@email.szu.edu.cn (D.L.); 2060453003@email.szu.edu.cn (Z.C.); dubin2016@email.szu.edu.cn (B.D.); zoumengqiang2020@email.szu.edu.cn (M.Z.); libozhe2019@email.szu.edu.cn (B.L.); xiongcong2018@email.szu.edu.cn (C.X.); jipeng_2013@163.com (P.J.); lczhang5354@szu.edu.cn (L.Z.); ygong@uestc.edu.cn (Y.G.); ypwang@szu.edu.cn (Y.W.); 2 Shenzhen Key Laboratory of Photonic Devices and Sensing Systems for Internet of Things, Guangdong and Hong Kong Joint Research Centre for Optical Fiber Sensors, Shenzhen University, Shenzhen 518060, China; 3 Guangdong Key Laboratory for Biomedical Measurements and Ultrasound Imaging, School of Biomedical Engineering, Health Science Center, Shenzhen University, Shenzhen 518055, China; tangshuo2020@email.szu.edu.cn (S.T.); xugaixia@szu.edu.cn (G.X.)

**Keywords:** Fabry–Pérot interferometer, breath sensor, micro-cantilever, two-photon polymerization, fiber sensor, wearable device

## Abstract

Respiration rate is an essential vital sign that requires monitoring under various conditions, including in strong electromagnetic environments such as in magnetic resonance imaging systems. To provide an electromagnetically-immune breath-sensing system, we propose an all-fiber-optic wearable breath sensor based on a fiber-tip microcantilever. The microcantilever was fabricated on a fiber-tip by two-photon polymerization microfabrication based on femtosecond laser, so that a micro Fabry–Pérot (FP) interferometer was formed between the microcantilever and the end-face of the fiber. The cavity length of the micro FP interferometer was reduced as a result of the bending of the microcantilever induced by breath airflow. The signal of breath rate was rebuilt by detecting power variations of the FP interferometer reflected light and applying dynamic thresholds. The breath sensor achieved a high sensitivity of 0.8 nm/(m/s) by detecting the reflection spectrum upon applied flow velocities from 0.53 to 5.31 m/s. This sensor was also shown to have excellent thermal stability as its cross-sensitivity of airflow with respect to the temperature response was only 0.095 (m/s)/°C. When mounted inside a wearable surgical mask, the sensor demonstrated the capability to detect various breath patterns, including normal, fast, random, and deep breaths. We anticipate the proposed wearable breath sensor could be a useful and reliable tool for respiration rate monitoring.

## 1. Introduction

Respiration rate (RR) is defined as the number of breaths per minute (bpm) and is a clinical sign that represents lung ventilation. RR normally ranges from 12 to 20 bpm for a healthy adult at rest [1]. RR has been determined as the vital sign that is most necessary to be continuously monitored [2]. It can reflect clinical information relating to neurological, cardiac, and pulmonary conditions. According to the guidelines from the National Institute for Health and Care Excellence of the United Kingdom in 2007, RR is the most sensitive parameter to detect any clinical deterioration.

There are various breath sensors for RR measurements, which can generally be classified as contact or noncontact. Contact methods refer to the cases in which the sensing system (or part of it) is directly attached to the subject’s body to detect signals including breath airflow, air temperature, humidity, specific components (e.g., CO_2_), and chest wall movements [3,4]. For noncontact methods, the instrument does not contact the subject, and uses radar, thermal, or optical-imaging-based monitoring solutions [5]. To date, electronic sensors are still the most mainstream of the commercialized breath sensors. However, these sensors are not capable of operating in strong electromagnetic environments due to the electronic transducers inside [6]. In fact, in situations associated with strong electromagnetic environments (e.g., in magnetic resonance imaging (MRI) systems), breath monitoring is still required for various purposes. These purposes include health monitoring, motion compensation, and clinical research such as functional MRI [7].

Fiber-optic sensors are attractive because they are immune to electromagnetic radiation. They also have the intrinsic advantages of flexibility, robustness, fast response, compact size, light weight, and remote sensing capability. Hence, fiber-optic sensors have recently attracted growing attention in applications for breath sensing, especially during MRI examination, as demonstrated in [8,9,10,11]. Among these fiber-optic sensors, fiber-optic-based flowmeters are an important category, and their principle of operation is based on the changes of coupled light power with fiber bending (or displacement) caused by the inhaled or exhaled airflow [12,13,14]. However, these types of sensors have the disadvantage of being sensitive to body motion artifacts, since the body motion of subjects also induces the macro-bending or displacement of fibers [3].

With the development of two-photon polymerization (TPP) microfabrication based on femtosecond (fs) laser, microstructures such as cantilever and clamped-beam can be directly fabricated at the end-face of optical fiber to form a micro Fabry–Pérot (FP) interferometer as a light-coupled microscale sensing platform. TPP microfabrication is a simple direct-writing technology and does not require an additional etching or coating process. Recently, our group demonstrated the applications of a micro FP interferometer on a fiber-tip for the measurement of nanoforce [15] and hydrogen [16] with high sensitivity and rapid response. These relevant works prove that the microcantilever is super sensitive to mechanical loading, with a detection limit down to tens of nanonewtons [15].

**Figure 1:**
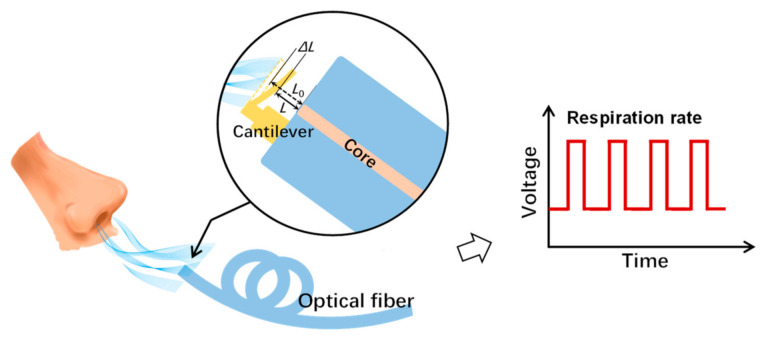
Schematic diagram of the proposed breath sensor. During the inhalation and exhalation cycles, the fiber-tip microcantilever deflects and recovers repeatedly. Thus, the respiration rate can be measured by recording the amplitude changes of the reflected light from the micro Fabry–Pérot interferometer formed by the microcantilever and the fiber end-face.

In this paper, a breath sensor based on a microcantilever fabricated on a fiber-tip is presented. The fiber-tip microcantilever was directly printed by TPP microfabrication, hence forming a micro FP interferometer. The RR signal was detected by demodulating the FP interferometer cavity length change induced by the bending of the microcantilever under the exhalation airflow, as shown in Figure 1. The airflow responses and temperature stability of the breath sensor were investigated. The real-time RR monitoring of different breath patterns was also demonstrated to prove the industrial applicability of the device.

## 2. Materials and Methods

### 2.1. Device Fabrication

**Figure 2:**
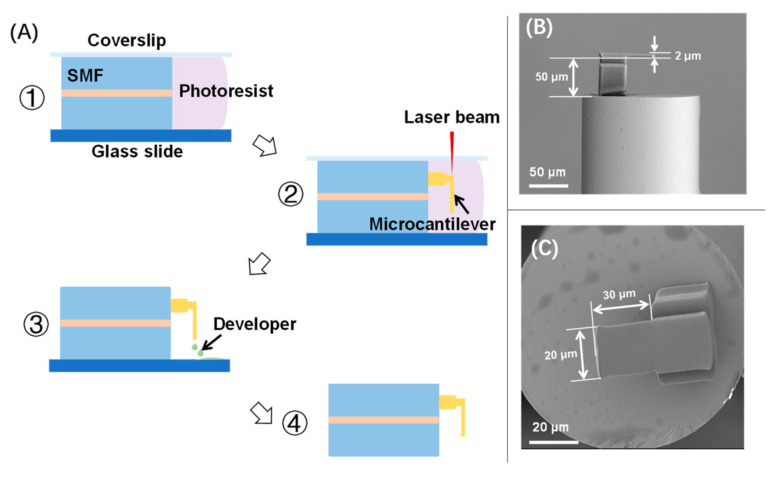
(**A**) Microfabrication process of a fiber-tip microcantilever by TPP technology based on fs laser; (**B**,**C**) are representative scanning electron microscopy (SEM) images of the fabricated device.

A commercial negative photoresist (PR) (Zhichu Optics Co., Ltd., Shenzhen, China) was applied for fabricating the fiber-tip microcantilever. The PR was composed of a photo-initiator (IGR-369, from Ciba Specialty Chemicals, Basel, Switzerland), monomers (SR444, SR368 and SR454, from Sartomer, Exton, PA, USA), a polymerization inhibitor (4-hydroxyanisole, MEHQ, from Sigma Aldrich, St. Louis, MO, USA), and an accelerator promoter (tetraethyl thiuram disulphide, TED, from Sigma Aldrich, St. Louis, MO, USA). The fabrication process has been described in detail elsewhere [15,16,17]. Briefly, a fiber-tip microcantilever was fabricated by TPP microfabrication based on femtosecond laser as shown in Figure 2A. Firstly, a precleaned single-mode fiber (SMF) was mounted in between a glass slide and a coverslip with its end-face immersed in a PR droplet. The polymerization of the microcantilever was then performed on a 3D air-bearing stage (Aerotech, Pittsburgh, PA, USA). The key parameters of the fs laser used in polymerization included a pulse width of 250 fs, a central wavelength of 1026 nm, and a pulse repetition rate of 200 kHz. To speed up the polymerization, the laser power was determined as 2 mW and the scanning velocity was determined as 300 μm/s. After polymerization, the unpolymerized PR was washed away by a developer of acetone and isopropyl alcohol (1:4, *v*/*v*). After washing and drying, the fiber-tip microcantilever was eventually fabricated. As shown in Figure 2B,C, the geometric parameters of the microcantilever were designed to be a length of 30 μm, a width of 20 μm, and a thickness of 2 μm. The cavity length of the fiber-tip FP interferometer was designed as 50 μm.

### 2.2. Reflection Spectrum Measurement

**Figure 3:**
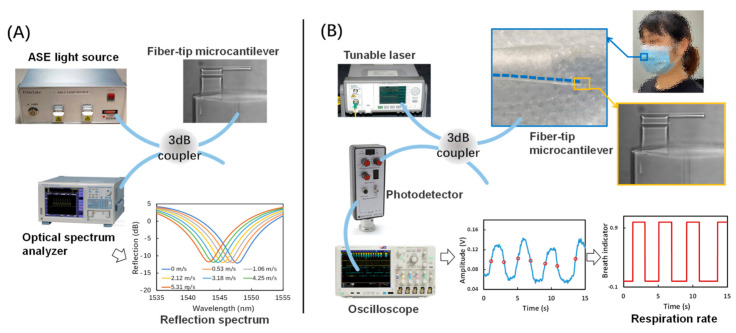
The experimental setups for (**A**) reflection spectrum measurement and (**B**) breath sensing.

The reflection spectrum from the micro FP interferometer formed by the fiber-tip microcantilever and the fiber end-face was measured using the setups shown in Figure 3A. An ASE (amplified spontaneous emission) light source (1250–1650 nm, Fiber Lake Co., Ltd., Shenzhen, China) was used to generate the input light of the micro FP interferometer, where a three-beam interference occurred with lights from the fiber-tip and the top and bottom surfaces of the microcantilever [16]. The following equation describes the function between the shift of dip wavelength (Δ*λ_r_*) and the cavity length reduction (Δ*L*) of the micro FP interferometer

Δ*λ_r_*/*λ_r_* = Δ*L*/*L* (1)

where *λ_r_* is the dip wavelength and *L* is the cavity length. An optical spectrum analyzer (AQ6317C, Yokogawa, Tokyo, Japan) was utilized to record the reflection spectrum signal.

### 2.3. Breath Sensing

The setup of breath sensing by measuring the RR is shown in Figure 3B. The intensity of the light reflected from the micro FP interferometer was monitored. The amplitude of this signal is a function of the FP interferometer cavity length *L* [18]. In this setup, input light from a tunable laser source (N7776C and N7778C, Keysight, Santa Rosa, CA, USA) was coupled to the micro FP interferometer through a 3 dB coupler. The wavelength of the input light was identified as corresponding to the −3 dB value upon the peak of reflection spectrum (∼1545 nm). The intensity of reflected light was then converted into an electric power signal and recorded by an oscilloscope (MDO3000, Tektronix, Beaverton, OR, USA). Due to its microscale size and excellent flexibility, the proposed breath sensor was able to be attached inside a disposable surgical mask, which was worn by the subject during the breath testing. Thus, when the subject breathed against the microcantilever, the corresponding RR signal could be collected from the oscilloscope and further processed by a dynamic-thresholding algorithm to generate the breath pattern.

## 3. Results and Discussion

### 3.1. Airflow Response

**Figure 4:**
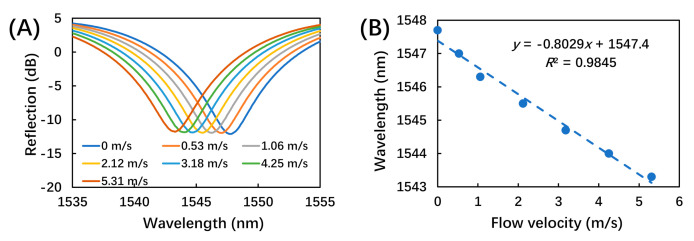
The responses of the breath sensor upon airflows at different flow velocities. (**A**) Reflection spectrum as a function of wavelength at different flow velocities; (**B**) linear fitting of the dip wavelength (∼1545 nm) as a function of flow velocity.

To measure the airflow response, the sensor was mounted in a silicone tube (3 mm in diameter), which was connected to a mass flow controller (MFC, Beijing Sevenstar Flow Co., Ltd., Beijing, China) controlling the dried nitrogen flow at a series of flow velocities from 0.53 to 5.31 m/s. This testing range covered the normal flow velocity of human breath of around 2 m/s [19,20]. Each flow velocity was applied for at least 5 min to ensure that the response of the breath sensor became stable. The evolution of the reflection spectrum as a function of applied airflow velocity was recorded. As shown in Figure 4A, the dip wavelength (∼1545 nm) of the reflection spectrum showed a blue shift (∼5 nm) with increasing flow velocity. This could be induced by the decrease of the interferometer cavity length due to bending of microcantilever under the aerodynamic loading. Figure 4B illustrates the linear fitting of the dip wavelength as a function of flow velocity, with a sensitivity of 0.8 nm/(m/s).

**Figure 5:**
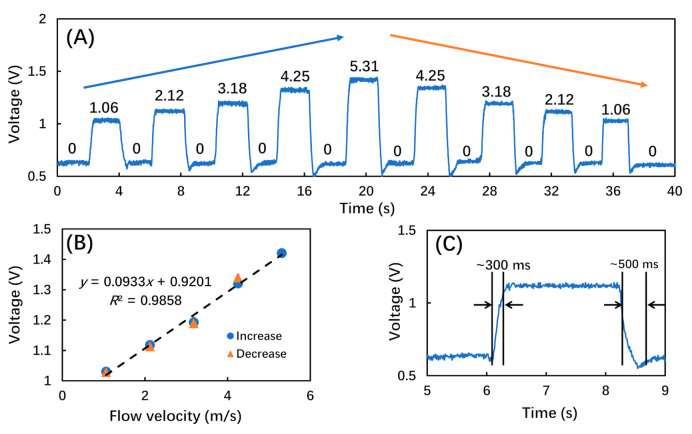
The responses of the breath sensor under airflow pulses at different flow velocities. (**A**) The amplitude of the reflected light under airflow pulses at different flow velocities (marked above the corresponding voltage data curves, unit: m/s); (**B**) linear fitting of output voltage data as a function of flow velocity; (**C**) a zoomed-in representative response under an airflow pulse of 2.12 m/s.

To mimic the periodic airflow of human breath, the MFC was also used to generate airflow pulses at a frequency of 15 times/min, which is within the normal range of respiration rate (12 to 20 bpm). In each airflow pulse cycle of ∼4 s, the MFC was first closed for ∼2 s, and then opened to generate airflow for ∼2 s at preset flow velocities from 1.06 to 5.31 m/s. The amplitude of the reflected light from the micro FP interferometer was then recorded, as shown in Figure 5. Figure 5A shows the voltage of the amplitude signal as a function of the flow velocities of applied airflow pulses. As the flow velocity first increased and then deceased, the output voltage value also increased and then decreased with almost the same frequency of input airflow pulses. This change was also due to the decrease of interferometer cavity length induced by the bending of the microcantilever under the aerodynamic loading. It was also noted that, due to the momentum of the microcantilever motion, there was a backward voltage when the microcantilever recovered from the aerodynamic-loading-induced bending, especially in the cases of larger flow velocities. Figure 5B plots the output voltage value as a function of the input flow velocity, with a linear fitting result showing a slope of 93 mV/(m/s). From the data in Figure 5B, a hysteresis error of less than 5% was calculated. Figure 5C shows a representative response under an airflow pulse, with a typical response and recovery time of ∼300 ms and ∼500 ms, respectively.

### 3.2. Temperature Stability

Considering the low melting point of the photoresist and the normal operation conditions of the breath sensor, the temperature response was tested from 25 to 55 °C. The sensor was placed in an oven with well-controlled temperature and the temperature was increased in steps of 5 °C in the tested range. Each temperature was maintained for 15 min to allow the sensor to reach a stable output. Then, both the evolution of reflection spectrum and the amplitude signal were recorded.

**Figure 6:**
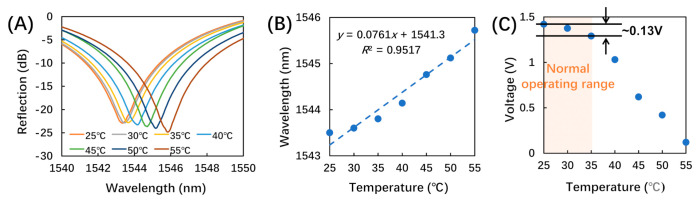
The temperature responses of the breath sensor. (**A**) Reflection spectra at different temperatures; (**B**) linear fitting of the dip wavelength (∼1545 nm) as a function of temperature; (**C**) amplitude of the reflected light under normal operating temperatures.

As shown in Figure 6A, the dip wavelength (∼1545 nm) of the reflection spectrum exhibited a red shift (∼2 nm) with increasing temperature. This was induced by the increase of the interferometer cavity length due to the heat-induced swelling of the photoresist [16]. Figure 6B illustrates the linear fitting of the dip wavelength as a function of temperature, with a sensitivity of 76.1 pm/°C. The cross-sensitivity of airflow with respect to temperature was calculated to be 0.095 (m/s)/°C, as the ratio of the two corresponding sensitivities shown in Figure 5B and Figure 6B.

Figure 6C shows the amplitude of the reflected light as a function of temperature. As a phototype demonstration, we constrained the application of the proposed breath sensor to be for indoor use at a room temperature of around 25 °C. Considering that body temperature is 37 °C, we determined the normal operation temperature as 25 to 35 °C [21]. Within this range, the output voltage only increased by 0.13 V as shown in Figure 6C, which was only ∼10.8% of the typical airflow response (∼1.2 V) to the flow rate of 2.12 m/s (the typical flow rate of human breath [19,20]). As a result, the impact of temperature variation under normal operating conditions was insignificant. Thus, both the low cross-sensitivity and the voltage change under normal operating temperatures indicate that the breath sensor has relatively low sensitivity to ambient temperature changes under normal operating conditions. However, there are many locations and situations with higher or lower temperatures, especially in outdoor settings, which would influence the performance of the proposed breath senor. Thus, future work to design and fabricate proper packaging for the breath sensor is needed to minimize the impacts from ambient temperature change.

### 3.3. Breath Sensing

The breath sensor was mounted in a disposable surgical mask to monitor human respiration, as shown in Figure 3B. During the testing, the subject was required to perform each of the breath patterns, including regular breath, deep breath, fast breath, and random breath. The corresponding time-dependent amplitude of the reflected light was recorded and processed by the dynamic thresholding algorithm to rebuild the breath pattern.

**Figure 7:**
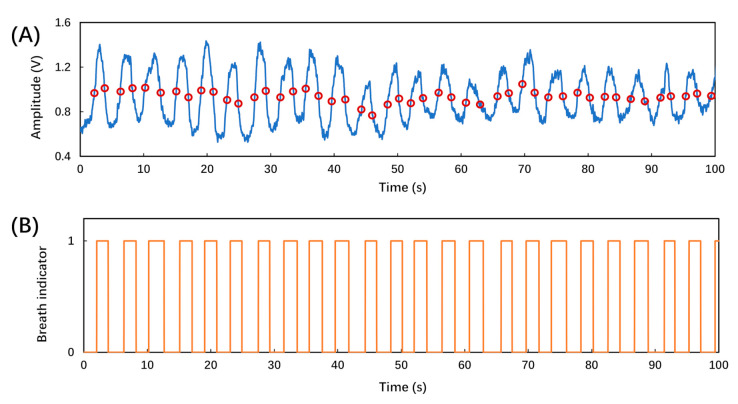
(**A**) Experimentally recorded breathing pattern using the breath sensor, in which the blue curve represents the time-dependent amplitude of the reflected light, and the empty red circles represent the dynamic thresholds in real time; (**B**) output of the breath analysis program using the dynamic thresholds corresponding to a regular breathing pattern.

Figure 7A shows the recorded amplitude signal of a regular breath lasting 100 s. During the exhale cycle, the breath from the subject’s nose induced a deflection in the fiber-tip microcantilever, which altered the amplitude signal. After it was converted to a voltage signal by the photodetector, the amplitude signal could be used to identify the inhalation and exhalation states. The baseline and amplitude signal showed certain fluctuations due to the turbulent flow within the space between the mask and the subject’s face, and the incomplete recovery of the fiber-tip microcantilever. Thus, a uniform threshold was not applicable for determining the inhalation and exhalation states.

**Figure 8:**
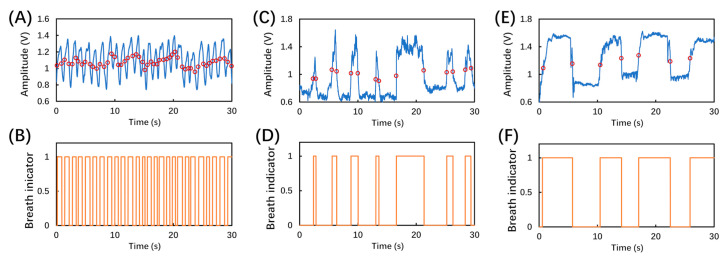
Experimentally recorded different breathing patterns and corresponding dynamic thresholds in real time for (**A**) fast breath, (**C**) irregular breath, and (**E**) deep breath; the output of breath analysis program using the dynamic thresholds corresponding to (**B**) fast breath, (**D**) irregular breath, and (**F**) deep breath patterns.

Instead, a dynamic threshold was applied to determine the inhalation and exhalation states [12,22,23]. Firstly, the “findpeaks” function in Matlab r2010b (MathWorks, Natick, MA, USA) was utilized to detect the peaks and valleys in the signal of time-dependent amplitude of the reflected light. There were multiple peaks in some of the signals, such as irregular breaths and deep breaths, as shown in Figure 8C,E. In the case of multiple peaks, we used the averaged values of the multiple adjacent peaks to represent the peak value. Secondly, we grouped each adjacent peak and valley as one pair to generate the threshold. Thirdly, the average values of all the adjacent peak–valley pairs were calculated as the dynamic thresholds, which were eventually used to determine the breath cycles, as shown in Figure 7A marked by empty red circles.

Figure 7B shows the breath indicator which reflects the breath cycles of inhalation and exhalation states according to the dynamic thresholds in Figure 7A. The breath indicator was determined as ‘0’ for amplitudes smaller than the dynamic threshold, which represented the inhalation state. On the other hand, the breath indicator was determined as ‘1’ for amplitudes larger than the dynamic threshold, which represented the exhalation state. As shown in Figure 7B, it is clear that the fiber-tip microcantilever-based breath sensor can be applied for monitoring regular breathing.

Additionally, in order to demonstrate that the sensor was also capable of monitoring the RR of various breath patterns, three more breathing behaviors were tested and recorded as shown in Figure 8. Figure 8A,C,E shows the recorded amplitude signals lasting 30 s of a fast, irregular, and deep breath, respectively, in which the empty red circles represent the dynamic thresholds in real time. Figure 8B,D,F shows the corresponding breathing patterns using the dynamic thresholds regarding fast, irregular, and deep breaths, respectively. Thus, the breath sensor was determined to also be capable of monitoring different types of breathing conditions.

During the tests, there were some incorrectly identified respiration signals, which were probably caused by the nonuniform airflow inside the disposable surgical mask. As the breath sensor was mounted inside the mask where the breath airflow was not well controlled, any factors that impact the breath airflow inside the mask would eventually impact the breath-sensing performance. To achieve more uniform breath airflow and reliable breath sensing, the breath sensor could further be integrated into breathing tubes, since these commercialized breathing tubes have a regular geometry and are capable of controlling the airflow direction. In addition, as an all-fiber device, the breath sensor is not only compact but also highly flexible and able to be integrated into oxygen masks, breathing tubes, etc. Due to its miniaturized size and high biocompatibility, it can even be placed close to the nose or lips with negligible discomfort and risks [3].

## 4. Conclusions

Here we proposed and experimentally demonstrated a novel wearable all-fiber breath sensor, which demonstrated excellent performance for monitoring human breath with different patterns. The sensing mechanism is based on the cavity-length reduction of a micro FP interferometer comprising a fiber-tip microcantilever and the end-face of a fiber, where the length reduction is caused by the mechanical bending of the cantilever under the exhaled airflow. Within the tested flow velocities from 0.53 to 5.31 m/s, the sensor achieved a high sensitivity of 0.8 nm/(m/s) and low cross-sensitivity of airflow with respect to the temperature response, of 0.095 (m/s)/°C. The proposed breath sensor also demonstrated an excellent capability for breath-pattern sensing by applying dynamic thresholds. Thus, the proposed sensor is thermally stable, compact, flexible, and wearable. We believe that it could be a promising key component of wearable RR monitoring devices, especially when using healthcare equipment associated with strong electromagnetic environments.

## Data Availability

The experimental data is contained within the article.
